# Temperature-Dependent Dynamics of Aβ42 and α‑Synuclein Monomers and Early Oligomerization of Aβ42: Shared Residues Mediate Intra- and Intermolecular β‑Sheets

**DOI:** 10.1021/acschemneuro.6c00242

**Published:** 2026-06-23

**Authors:** Gabriel F. Martins, Cristiano Rocha, Nuno Galamba

**Affiliations:** BioISI - Biosystems and Integrative Sciences Institute, Faculty of Sciences of the University of Lisbon, C8, Campo Grande, Lisbon 1749-016, Portugal

**Keywords:** hydrophobic effect, oligomerization, neurodegenerative diseases, molecular dynamics

## Abstract

We used molecular dynamics simulations to investigate how temperature modulates hydrophobic interactions and β-sheet formation in the CHARMM36m model of intrinsically disordered proteins, focusing on a monomer of amyloid-beta (Aβ42) and α-synuclein, as well as a dimer and tetramer of Aβ42. For the isolated monomers, increasing temperature leads to an increase in intramolecular contacts, promoting hydrogen bonding and secondary-structure reorganization toward β-sheet and turn motifs. Analysis of the dimer and tetramer of Aβ42 reveals increased conformational heterogeneity at high temperatures, suggesting a smaller-than-expected configurational entropy penalty upon association. Thus, whereas monomers undergo temperature-induced compaction, enhancing intramolecular interactions, including the formation of β-sheets, in the dimer and tetramer, hydrophobic stabilization is redirected toward aggregation, promoting cross-β-sheet formation, peptide elongation, and the emergence of spherical conformations. Notably, the residues that stabilize intramolecular β-sheets in the monomer (approximately sequences 16–22 and 29–36) largely overlap with those that form cross-β-sheet motifs in the aggregates, suggesting that intramolecular β-sheet formation is intrinsically linked to aggregation propensity. These results, in close agreement with earlier NMR measurements of the monomer and protofibrils, reveal a competition between intra- and intermolecular hydrophobic interactions, with intermolecular interactions ultimately becoming more favorable than the intramolecular interactions that stabilize monomer solvation.

## Graphical abstract

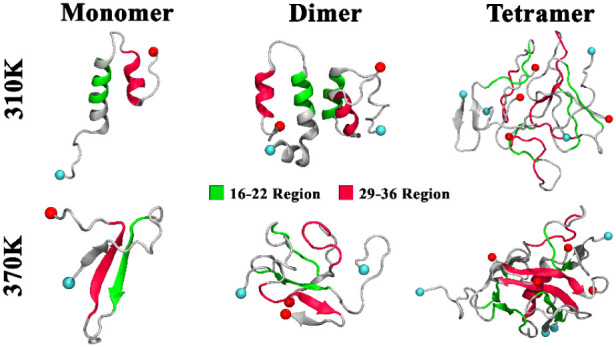

## Introduction

Amyloid aggregates, especially prefibrillar soluble oligomeric species,^1–5^ are believed to be implicated in the etiology of neurodegenerative diseases such as Alzheimer’s^6,7^ and Parkinson’s disease.^8,9^ Although the underlying mechanisms of these diseases remain incompletely understood and multifactorial hypotheses have been proposed,^10–12^ the oligomeric hypothesis^1,7,13^ remains a central framework for understanding their pathogenesis.

From a molecular perspective, hydrophobic interactions are believed to be the main thermodynamic driving force for protein folding and aggregation,^14–18^ whereas the formation of backbone inter-β-strand hydrogen bonding between amide and carbonyl groups (NH···O=C) gives rise to the cross-β motif that characterizes both pathogenic and functional amyloids.^19,20^ This motif involves the stacking of β-sheets formed by β-strands running perpendicular to the fibril axis.

Although distinct amyloid fibrils formed by the same protein have been identified as a result of amyloid polymorphism,^19,21–29^ these are all characterized by cross-β motifs. Additionally, various amyloid-beta (Aβ)^30–40^ and α-synuclein (α-syn)^8,41–44^ oligomers were also reported in the literature. Notably, some oligomers do not exhibit cross-β sheets,^37,45^ whereas others support a neurotoxicity mechanism consistent with the oligomeric pore hypothesis, in which oligomers disrupt membrane integrity by forming pore-like structures within the neurons’ membrane.^22,35,36,41,46,47^

Experimental studies long showed that intrinsically disordered proteins (IDPs) generally become more compact and less disordered as the temperature is raised.^48–50^ This behavior suggests that hydrophobic interactions govern these conformational transformations. However, Schuler and coworkers also showed that the most pronounced compaction among several IDPs was observed for the least hydrophobic protein, proposing that solvation of the most hydrophilic residues significantly contributed to the collapse.^48,50^ Uversky and Fink showed that a temperature increase (or pH decrease) induced the transformation of α-syn into a partially folded conformation.^49,51^ This conformation was proposed to be aggregation-competent and, therefore, a primary step in the fibrillation of α-syn. Ferrone also proposed that an initial unfavorable conformational transition of the Aβ monomer could constitute the rate limiting step for nucleation with these monomers acting as nuclei.^52^

The above-mentioned temperature-induced compaction contrasts with the melting of globular proteins, showing that heat induces the breakdown of hydrogen bonds (HBs) associated with the secondary structure (e.g., α-helices and β-sheets) of globular proteins while inducing the formation of HBs linked to the formation of secondary structure motifs in IDPs. As expected, an increase in temperature induces a similar compaction in denatured proteins, primarily but not exclusively^50^ associated with the strengthening of the hydrophobic effect.^50,53^ Thus, even though globular proteins unfold with increasing temperature, the hydrophobic effect is expected to promote their collapse into compact, spherical-like disordered conformations.^53^ Whereas protein folding at high temperatures is not favorable because of the large configurational entropy decrease involved, a structureless compaction does not involve such a pronounced entropic reduction.^48^ This, in addition to the fact that the hydration free energy of both hydrophilic and hydrophobic groups increases (less negative and more positive, respectively) explains this compaction even in amphiphilic molecules such as proteins.^48,54^

Notwithstanding the temperature-modulated compaction in IDPs may arise from several factors linked to their amphiphilic nature, the hydrophobic effect is expected to play a dominant role.^18,55–57^ The hydrophobic effect increases with the temperature for small solutes and groups,^57,58^ manifesting in an increase of the hydration free energy and of the aggregation driving force, through the loss of water molecules upon aggregation, increasing the configurational entropy of water molecules. A structural compaction is seen for long hydrocarbons, resulting in an increase of the solvation entropy and enthalpy above those for small solutes, at temperatures below *T*_*S*_, the temperature at which entropy is zero.^59^ However, hydrophobic interactions in peptides and proteins are complicated by their amphiphilic nature, and the relationship between hydrophobic interactions and the onset of intramolecular β-sheet in monomers and intermolecular β-sheet formation in dimers and larger oligomers, remains elusive. Molecular dynamics (MD) simulations provide a complementary framework to experiments for investigating these transformations at the molecular level. The major limitation arises from sampling limitations, since the formation of β-sheet structures is expected to occur on time scales of μs,^60^ longer than the hydrophobic collapse.^53^ However, aggregation as well as the formation of cross-β-sheet structures should occur on a faster time scale at high temperatures. Various simulation studies investigated the effect of temperature on the structure of some IDPs aiming at assessing structural changes in the monomer.^61–66^ The way temperature modulates aggregation and its relationship with the monomer transformations, in particular, the interplay between the compaction of the monomer and the intermolecular interactions and elongation of some oligomers and protofibrils, remains, however, poorly understood.

Here, we investigated through all-atom MD the effect of temperature on the conformational space of Aβ and α-syn monomers and the oligomerization of Aβ, to gain insight into the relationship between the hydrophobic effect, the formation of intramolecular β-sheets in the monomer and cross-β-sheets in the oligomers.

## Methods

Molecular dynamics (MD) simulations of α-syn and Aβ42 models in 0.1 M NaCl aqueous solutions were performed in the isothermal–isobaric (*NpT*) ensemble in a cubic box with periodic boundary conditions. The protein and the peptide were modeled with the CHARMM36m^67^ force field and water was modeled with the TIPS3P model. The starting conformation of Aβ42 used to simulate the monomer and the dimer was obtained from the study of Tomaselli et al.^63^ (PDB: 1Z0Q) while the initial structures for the tetramer were constructed from a monomer extracted from a fibrillar assembly (PDB ID: 2MXU).^68^ The starting conformation of α-syn was a monomer from a fibril (PDB: 2n0a).^69^ The 1Z0Q structure has 0% β-sheet, 12% bend, 26% α-helix, 38% turn, and 12% random coil, whereas the monomer from the 2MXU has 0% β-sheet, 16.7% bend, 21.4% α-helix, 0% turn, and 61.9% random coil as determined using the DSSP algorithm^70^ (see below). The initial α-helical content in the 2MXU monomer arises from residues 1–10, which are absent in the original PDB structure and were modeled as an α-helix. Although somewhat arbitrary, the latter was motivated by the need to avoid excessively large simulation boxes associated with fully extended conformations, while also minimizing any bias toward β-sheet formation in the initial structure. The monomer of 2n0a has 0% β-sheet, 17% bend, 4% α-helix, and 70% random coil. The overall choice of these structures both for Aβ42 and α-syn was primarily driven by the absence of β-sheet, thus avoiding any potential bias toward preformed aggregation-prone structures. To model early oligomer formation, simulations were initiated from systems containing either two Aβ42 monomers with a center-of-mass distance between their central amino acids of *d*_*ca*_ > 40 Å, or four monomers positioned such that *d*_*ca*_ > 50 Å between any pair of peptides.

All MD simulations were performed with the program GROMACS.^71^ The systems were studied at 310 and 370 K and 0.1 MPa. The choice of the latter temperature was motivated by the expectation that a hydrophobic collapse and especially the formation of cross-β motifs in the dimer and tetramer could be observed within accessible (∼1 μs) simulation times. A monotonic increase of protein compaction was observed for almost all proteins studied by Wuttke et al.^48^ up to 350 K, and up to 360 K for α-syn.^49^ The temperature and pressure were controlled using the Nosé–Hoover thermostat,^72,73^ and the Parrinello–Rahman barostat,^74^ respectively. The equations of motion were integrated with the leapfrog Verlet algorithm using a 2 fs time step. Long-range electrostatics were treated with the particle-mesh Ewald (PME)^75^ method, with a 1.0 nm cutoff for both van der Waals interactions and the real-space component of PME. Heavy atom–hydrogen covalent bonds were constrained using LINCS.^76^

Energy minimization was performed using the steepest-descent algorithm, followed by a 50 ps *NVT* equilibration and a subsequent 50 ns *NpT* equilibration. The trajectories were then propagated in the *NpT* ensemble for 500 ns for the monomers at 310 and 370 K, and the dimer at 310 K; for the Aβ42 dimer at 370 K the simulations were extended up to 1 μs for reasons discussed herein. Five and three independent replicates were carried out both for the monomer and dimer of Aβ42 at 310 and 370 K, respectively. For the α-syn monomer, four replicates were performed at both temperatures. For the Aβ42 tetramer, three independent replicates were performed at 310 K. Two trajectories were propagated up to 500 ns and one to 1 μs. However, only two trajectories (a 500 ns and a 1 μs trajectory) in which the tetramer was formed were included in the analysis. At 370 K, three replicates were performed, two 500 ns trajectories and one 1.5 μs trajectory. In this case, all trajectories were included in the analysis. Although all the above-mentioned trajectories for all systems were initially propagated for 500 ns, some were subsequently extended to ensure that the observed behaviors were not transient effects resulting from insufficient sampling. Trajectory frames were saved every 0.2 ps for the monomer and tetramer simulations.

Principal component analysis (PCA)^77^ (aka essential dynamics) was used for linear dimensionality reduction, combined with hierarchical density-based spatial clustering of applications with noise (HDBSCAN),^78^ to characterize the dominant conformational states of each system. PCA transforms the atomic coordinates from MD trajectories into a set of orthogonal eigenvectors known as principal components (PCs) that are ordered by decreasing structural variance. Since Aβ42 and α-syn are intrinsically disordered, a broad distribution of conformations is expected, leading to multiple PCs with significant contributions to the overall variance.^50^ Here, the dimensionality was restricted to the first two PCs, which represent the most relevant uncorrelated collective motions of the protein or peptide. The latter account for the largest conformational variance, 37% and 35% for the monomer of Aβ at both 310 and 370 K, and 67% and 32% for the α-syn monomer at 310 and 370 K, respectively, with subsequent components capturing progressively smaller fluctuations. We stress that much larger percentages (∼70%–90%) are often found for globular proteins.^77,79,80^ Similar values were observed for the first ten PCs, specifically, 84% and 80% for Aβ42 and 91% and 75% for α-syn monomer at 310 and 370 K. For the Aβ dimer the first two PCs accounted for 54% at 310 K and 27% (500 ns trajectory) and 29% (1 μs trajectory) at 370 K; the latter results indicate convergence.^81^ Notice, these results already demonstrate an increased conformational variance at high temperatures, especially for the monomer of α-syn and the dimer of Aβ.

PCA was performed using the non-mass-weighted covariance matrix of C_α_ atomic displacements, yielding a 126 × 126 covariance matrix for Aβ42 (3 Cartesian dimensions × 42 residues) and a 420 × 420 covariance matrix for α-syn (3 Cartesian dimensions × 140 residues); further details were provided elsewhere.^82^ To further dissect the conformational ensemble, HDBSCAN clustering was applied to the PCA-projected coordinates. This density-based hierarchical algorithm identifies regions of high local point density separated by low-density boundaries, allowing the detection of clusters with arbitrary shape and no requirement to predefine the number of clusters. This makes HDBSCAN particularly suitable for intrinsically disordered proteins, whose conformational ensembles are heterogeneous, non-Gaussian, and often characterized by diffuse, partially overlapping basins. HDBSCAN operates on pairwise distances between data points in the reduced PC space, using the Euclidean distance between their PCA coordinates as the similarity metric. Local density is estimated through the core distance of each point, defined as the distance from point *x* to its *k*th nearest neighbor,^78,83,84^

1 $$core_{k}\left(x\right)=\mathrm{distance}\left(x,\;k^{th}\;\mathrm{nearest}\;\mathrm{distance}\right)$$

These core distances are used to compute the mutual reachability distance (mrd) between two points *a* and *b*,^78,83,84^

2 $$d_{mrd-k}\;\left(a,\;b\right)=\max\;\left\{core_{a},\;core_{b},\;d\left(a,\;b\right)\right\}$$

where *d*(*a*, *b*) is their Euclidean distance. This metric effectively couples density and distance, promoting the formation of compact clusters while filtering sparse or transitional regions as noise. Using mutual reachability distances, HDBSCAN constructs a minimum spanning tree (MST) using Prim’s algorithm,^85^ capturing the hierarchical density connectivity of the data set. Clusters are extracted by progressively condensing the MST across multiple density levels and selecting the most persistent branches. Cluster stability is quantified as the persistence of a cluster across these density thresholds, serving as a measure of structural robustness within the conformational ensemble. The minimum cluster size parameter determines the smallest set of points required to form a statistically meaningful cluster, thereby defining the density threshold for cluster survival. In light of the heterogeneous nature of IDPs, this parameter was set to 1% or even 0.1% of the sampled frames, allowing detection of minor yet structurally relevant substates while avoiding spurious fragmentation into noise. Trajectory frames were subsampled using a consistent stride for the PCA/HDBSCAN analysis. This resulted in ∼12500 structures for the Aβ42 monomer at 310 and 370 K. For α-syn ∼10000 structures were analyzed at both 310 and 370 K. For the Aβ42 dimer systems, ∼5000 structures were analyzed at both temperatures for the 500 ns trajectories. For the longer (1 μs) trajectory the number of analyzed structures increased to ∼11000.

Additionally, the conformational landscapes of the monomers of Aβ42 and α-syn were characterized using two-dimensional free-energy surfaces (FES) constructed along selected collective variables (CVs). The FES were obtained from the joint probability distributions of the CVs, and the resulting maps represent the relative populations of conformational states, where low-free-energy basins correspond to high-probability, thermodynamically favored conformations. The two collective variables considered were the radius of gyration (*R*_*g*_) and the solvent-accessible surface area (SASA). Intramolecular interactions were further analyzed by calculating HBs and residue–residue C_α_ distance maps. A similar approach was carried out for the intermolecular distances between the two Aβ42 peptides in the dimer. The secondary structure of the Aβ42 and α-syn as well as the intermolecular β-sheet content of each system was assessed using the program DSSP (Dictionary of Secondary Structure of Proteins)^70^ which uses a strictly energetic hydrogen-bond definition (*E*_HB_ < −0.5 kcal/mol).

## Results and Discussion

The hydrophobic effect is influenced both by the pH and temperature.^49,57,58,86^ For IDPs such as α-syn and Aβ42, hydrophobic interactions result in a compaction of the monomer while seemingly favoring the self-assembly into oligomers implicated in disease.^48,51^ Here, we analyzed the behavior of these monomers at 310 and 370 K to gain insight into how hydrophobically driven structural transformations in the monomer may relate to the formation of oligomers and fibrils composed of cross-β sheets.

### Aβ42 and α-Syn Monomer

Figure 1 shows extensive structural analysis for monomeric Aβ42 at both temperatures (i.e., 310 K and 370 K).

**Figure 1:**
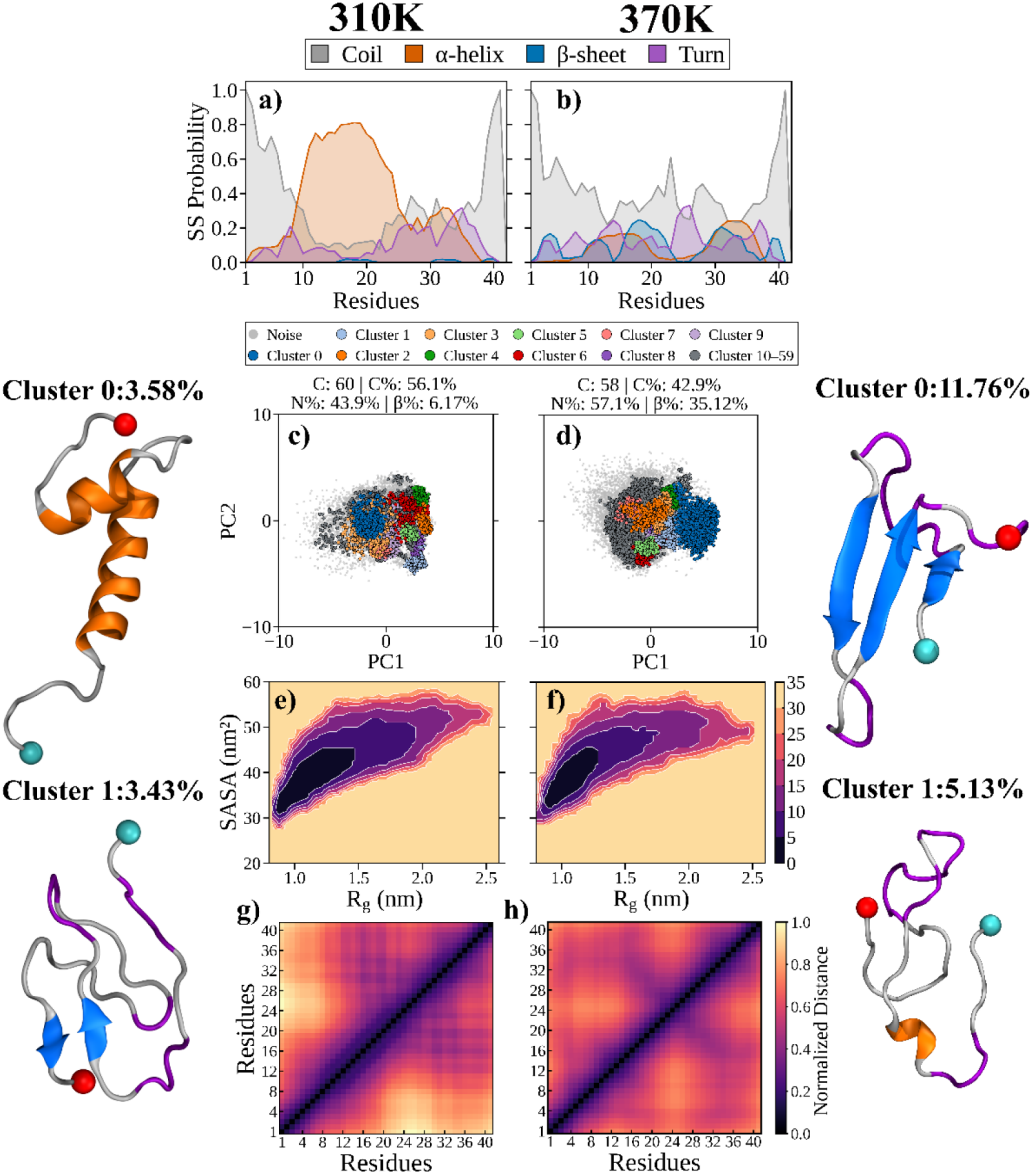
Aβ42 monomer structural properties at 310 and 370 K. (a, b) Secondary structure probabilities; (c, d) PCA atomic displacement projections in the plane formed by the first two eigenvectors coupled with HDBSCAN analysis and medoid representations of the two most populated clusters (clusters 0 and 1) using 1% of the number of frames as minimal cluster size; HDBSCAN parameters are displayed above the plots (C—number of clusters, C%—percentage of frames within clusters, N%—percentage of frames within noise, β%—percentage of frames containing β-sheet structure); (e, f) reduced FES in kJ/mol calculated using *R_g_* and SASA; (g, h) intramolecular C_α_ distance maps: the distances were normalized by the largest average distance between a pair of residues. The latter was found for the trajectory at 310 K (∼2.89 nm).

The secondary structure distributions (Figure 1a and b) reveal a significant decrease in α-helix propensity with the temperature increase, as expected, along with a broader increase in β-sheet content, including in the central hydrophobic region (residues 16–22 (KLVFFAE)), pivotal for aggregation,^29,87,88^ as well as in the region 29–36 (GAIIGLMV). These sequences were found, through combined MD and NMR data, to form a hairpin in Aβ42, but not in Aβ40.^89^ Thus, temperature evidence a favoring of the typical β-sheet amyloid-like structures, *albeit* intramolecular. Whether the residues involved in these intramolecular β-sheets become involved in intermolecular β-sheets in the dimer and tetramer is discussed in the next sections. Additionally, a population of turn conformations was detected around residue 27 at 370 K, connecting the β-sheet regions around the central core (∼16–22) and C-terminal (∼29–36), in close agreement with the results of Ball et al.^89^ Notably, a similar β-strand-turn-β-strand sequence encompassing residues 18–42 with the turn in the 27–30 region was found in Aβ42 fibrils through NMR.^90^ The latter study showed that intermolecular side-chain contacts are formed between the odd-numbered residues of strand β1 of the *n*th peptide and the even-numbered residues of strand β2 of the (*n* – 1)­th peptide. This suggests that intramolecular β1−β2 interactions within a monomer compete with intermolecular β1−β2 interactions that stabilize stacked, parallel, in-register β-sheets between monomers.^90^ We anticipate that our results support this competitive balance.

PCA analysis suggests a slightly greater conformational variability at 370 K, consistent with a more dynamic system at higher temperatures. However, for a short IDP the differences in projected variance remain limited. The HDBSCAN clustering analysis (Figure 1c and d) supports this result exhibiting an increase in the percentage of noise conformations, 57.1% at 370 K system in opposition to 43.9% at physiological temperature, although a similar number of clusters was found at both temperatures. However, the most prominent result is the increase of β-sheet structure in the dominant high-temperature conformation (cluster 0), in contrast with the most populated cluster at 310 K. Thus, an overall increase in the fraction of β-sheet-containing conformations across all clusters is observed, rising from 6.2% at 310 K to 35.1% at 370 K.

The FES (see Figure 1e and f) support a mild compaction of Aβ42 with a reduction in *R*_*g*_ at 370 K, while the SASA remains largely unaltered, although some reduction can be seen for the more extended conformations (larger *R*_*g*_). Figure 1g and 1h also show a noticeable increase in intramolecular contacts at 370 K resulting in a considerable reduction in long “contacts” (represented by the longest distances at yellow). This is consistent with an enhancement of the intramolecular hydrophobic interactions promoting structural rearrangements toward more compact, β-sheet enhanced conformations, reported in some previous works.^48,62,64,66,91,92^ A similar contact map for the monomer of Aβ42 was reported by Chen et al.^93^ (see Figure 2a of ref 93) using the same force field and starting from a 3 μs MD simulation of monomeric Aβ42. Thus, the same short distance (dark blue) region is observed between, approximately, sequences 16–22 and 29–36.

**Figure 2:**
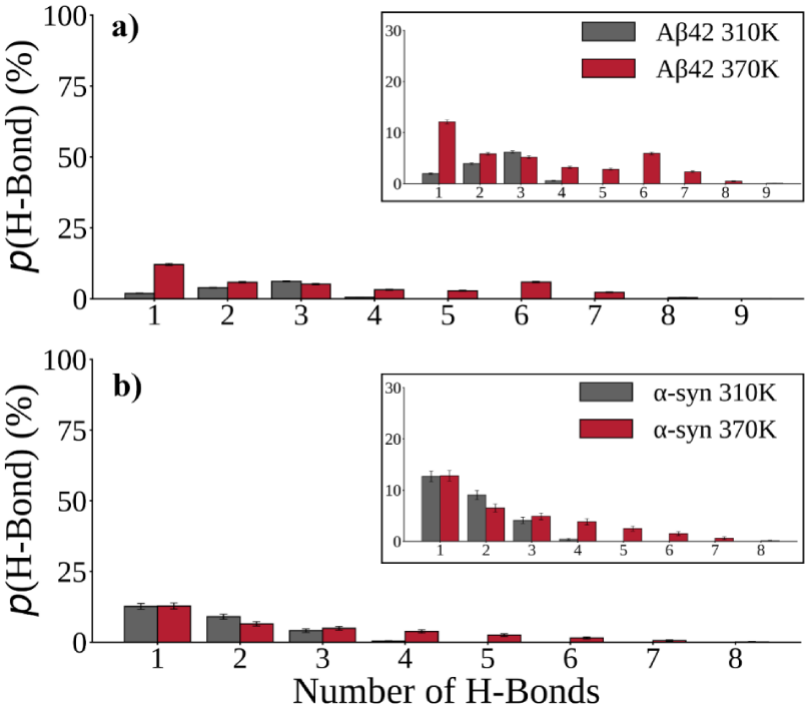
Probability of finding a conformation with *N*_HBs_ between (a) the sequences 16–22 and 29–36 of Aβ42 at 310 and 370 K, and between (b) the 85–95 and the 110–120 sequences of α-syn at 310 and 370 K. The contacts between the sequences 16−22 and 29−36 in Aβ42 were found to form a hairpin in Aβ42, but not in Aβ40.^89^ These contacts have been linked with the larger aggregation propensity of Aβ42 relative to Aβ40. The contacts between the sequences 85–95 and the 110–120 sequences have been linked with α-syn’s monomer autoinhibitory aggregation conformations.^94^ Error bars are standard deviations of the probability, $\sigma =\sqrt{p(1-p)/N}$ , where *p* is the probability of observing a frame with *N*_HBs_.

These structural changes also suggest an increase in intramolecular HBs. Figure 2a displays the probability of finding a conformation with *N*_HBs_ between the sequences 16–22 and 29–36. A clear increase is seen in the number of frames with *N*_HBs_ > 3 at 370 K compared to 310 K.

Figure 3 shows a similar analysis for the monomeric state of α-syn. A more pronounced pattern, consistent with the enhancement of hydrophobic interactions at high temperatures is observed, compared to Aβ42. The secondary structure (Figure 3a and b) shows a decrease in coil and an increase in β-sheet content, although less marked than for Aβ42, across the whole protein with particular prominence in the pre-NAC region (NAC comprises the sequence 61–95), encompassing the P1 (residues 36–42) and P2 (residues 45–57) sequences,^95^ also believed to play a central role in aggregation.

**Figure 3:**
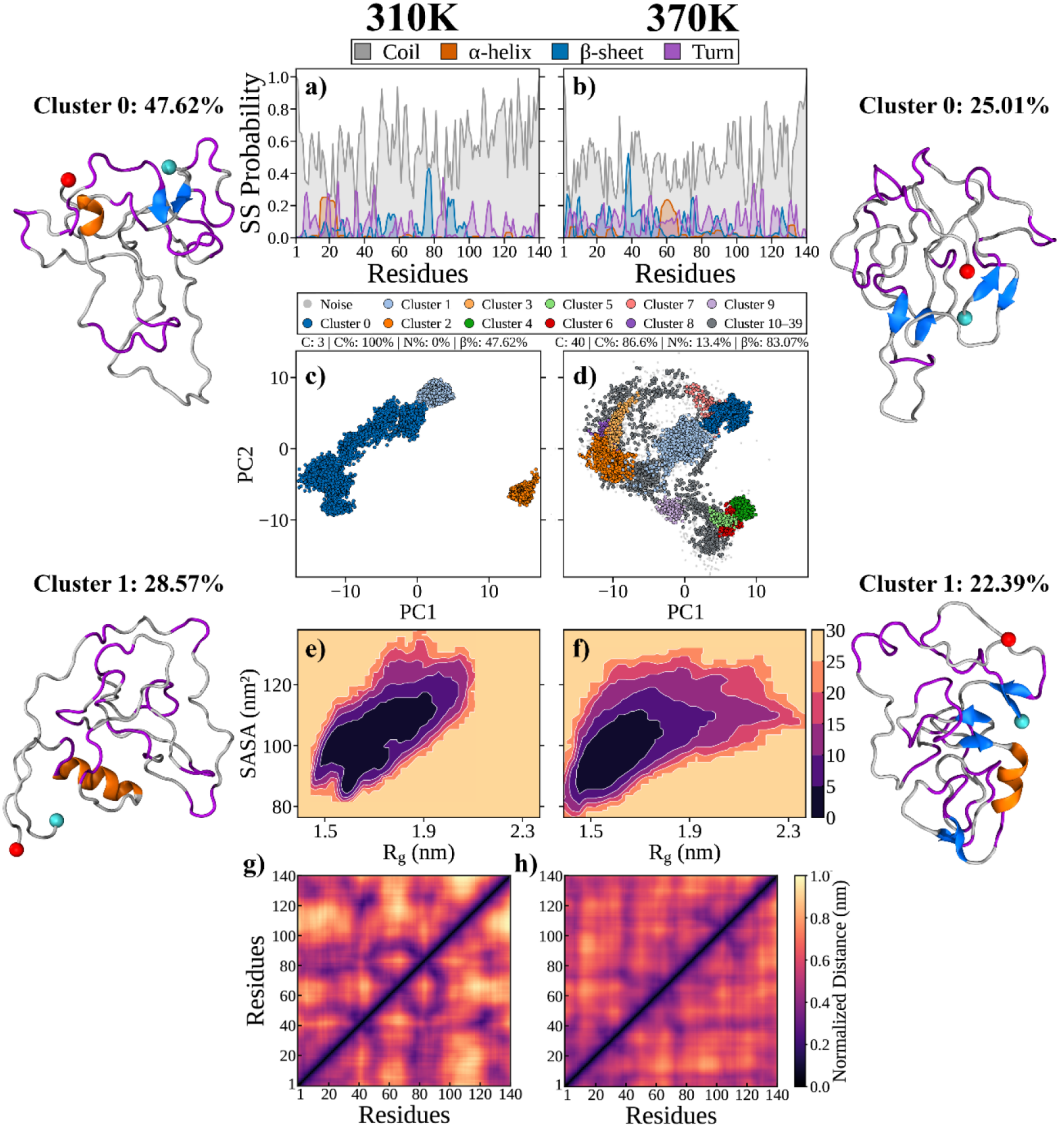
α-syn monomer structural properties at 310 and 370 K. (a, b) Secondary structure probabilities; (c, d) PCA atomic displacement projections in the plane formed by the first two eigenvectors coupled with HDBSCAN analysis and medoid representations of the two most populated clusters (clusters 0 and 1) using 1% of the number of frames as minimal cluster size; HDBSCAN parameters are displayed above the plots (C—number of clusters, C%—percentage of frames within clusters, N%—percentage of frames within noise, β%—percentage of frames containing β-sheet structure); (e, f) reduced FES in kJ/mol calculated using *R*_*g*_ and SASA; (g, h) intramolecular C_α_ distance maps; the distances were normalized by the largest average distance between a pair of residues. The latter was found for the trajectory at 310 K (∼3.99 nm).

PCA and HDBSCAN analysis reveals a substantial increase in conformational dynamics at 370 K, represented in Figure 3c and d. HDBSCAN clustering shows that the percentage of structures with β-sheet conformations increases to 83.1%, meaning that almost all conformations show at least some residues forming intramolecular β-sheets, whereas at 310 K only 47.6% of the conformations present this type of structure. The number of clusters, in turn, increases from 3 at 310 K to 40 at 370 K whereas noise increases from 0% to 13%.

The FESs (Figure 3e and f) show a reduction of the *R*_*g*_ at 370 K, in the lowest free energy basin, although a broader interval of *R*_*g*_ can be seen, supporting increased conformational dynamics. A more modest SASA decrease is also observed.

An increase in intramolecular contacts between the NACterm sequence (residues 85 to 95) and some residues in the C-terminal domain are also observed at high temperatures (Figure 3h). These intramolecular contacts are important as they have been suggested to preclude aggregation of α-syn.^94^ An increase in the number of HBs between the 85–95 and the 110–120 sequences^94^ of α-syn at 310 and 370 K can be seen in Figure 2b. These results show that, like Aβ42, temperature drives α-syn toward more compact, β-sheet-rich, and intramolecular HB-stabilized conformations.

### Aβ42 Dimerization

We now discuss the effect of temperature on the dimerization of Aβ42. While hydrophobic interactions are thought to be the main driving force for protein aggregation these also promote the formation of intermolecular HBs, like intramolecular HBs in the monomer. Aggregation implies, therefore, the replacement of intramolecular by intermolecular interactions, involving some dewetting^96^ next to hydrophobic and possibly some hydrophilic residues involved in intermolecular HBs.

The formation of Aβ42 dimers was identified using a distance-based criterium to map the number of contacts along time (Figure S1). Aggregation onset was probed by assessing intermolecular atomic contacts within 0.5 nm between the Aβ42 peptides. This was applied both to all the residues pairs and to the 16–22 amyloidogenic core sequence. Figure S1 shows that the number of such contacts increases over time and that dimerization occurs on average at a faster time scale (as expected) at 370 K. This was also verified through visual inspection of the trajectories. The use of shorter and longer intermolecular atomic contact definitions leads to similar results (see Figure S2). A decrease in the average distance between the center-of-mass of the backbone atoms of residues 16–22 in the two peptides is also observed (see Figure S2).

The time-windows where the number of contacts, *N*_c_, is below and above a threshold (i.e., *N*_c_ < 20 and *N*_c_ ≥ 20) (see Figure S1) were used, respectively, in the analysis of the separated monomers (unbound state) and the monomers-in-the-dimer structure (bound state). Additionally, we monitored the time evolution of the α-helix and β-sheet along with the *R*_*g*_.

Figure 4 shows that the α-helix decreases and the β-sheet and *R*_*g*_ increase upon dimerization. This effect is more pronounced at 370 K, suggesting that, rather than the isolated monomers becoming more compact as the temperature increases, hydrophobic-induced aggregation is stabilized through β-sheet formation, leading to increased elongation of the monomers in the dimer (see also the intra- and intermolecular contact maps; Figure S4).

**Figure 4:**
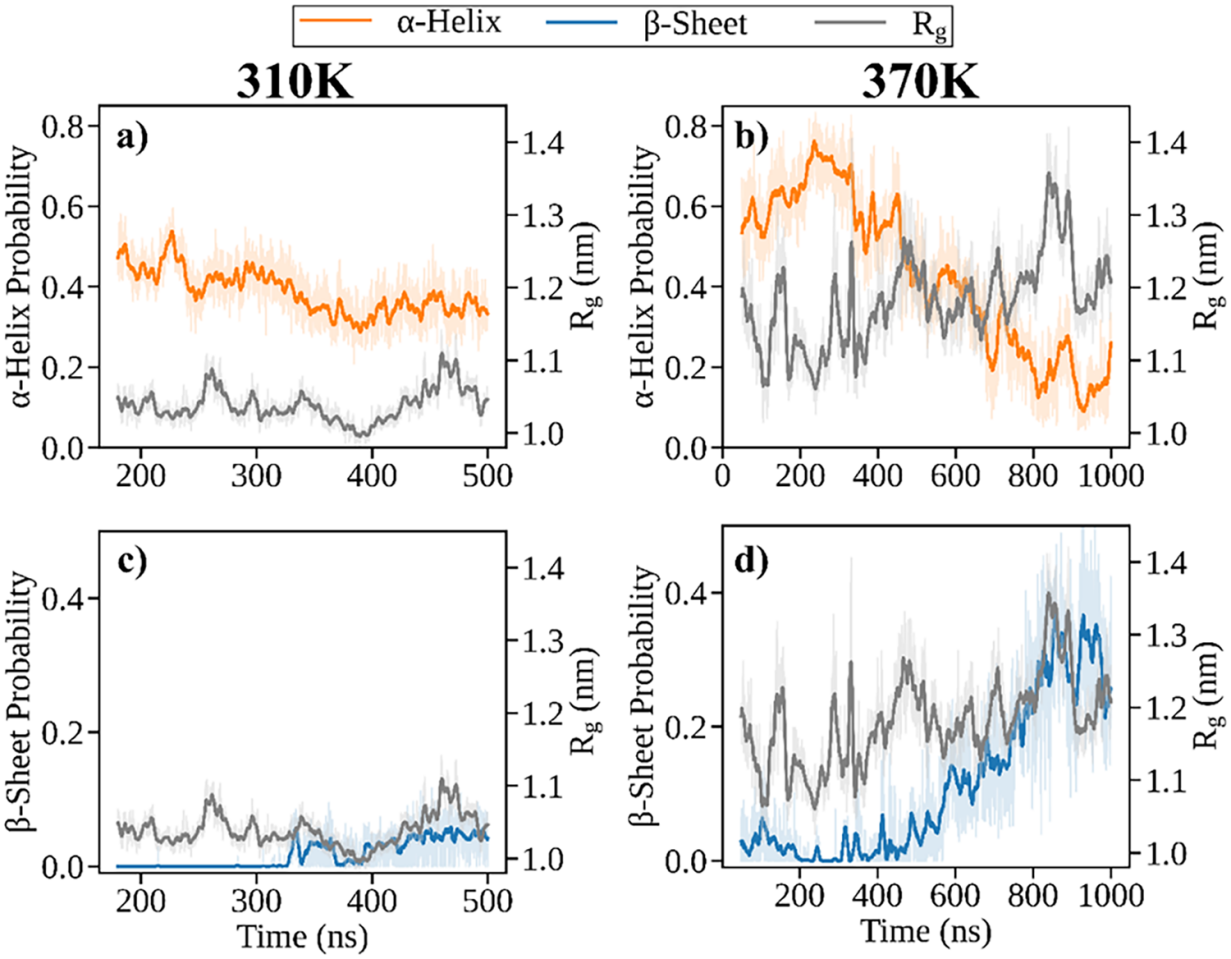
Time evolution of the α-helix and *R_g_* of the monomers in the dimer of Aβ42 at (a) 310 K and (b) 370 K; time evolution of the intramolecular β-sheet and *R_g_* of the monomers in the dimer of Aβ42 at (c) 310 K and (d) 370 K. The analysis was restricted to the time intervals in which the two monomers are bound: 180–500 ns at 310 K and 80 ns–1 μs at 370 K. Results are averages over the two monomers and the three replicates on a common time-window corresponding to the latest onset among the replicates—β-sheet does not include cross-β-sheet.

A decrease in α-helix and a gain in intramolecular β-sheet and turn structures, including in residues 16–22, can also be seen in Figure 5. Again, this is more pronounced at *T =* 370 K and for the longer simulations (Figure 5g and h). However, the most striking result is the appearance of intermolecular β-sheets around the 16–22 region at 370 K (Figure 5f and i). These results indicate that the same regions are involved in both intramolecular (see Figure 1b) and intermolecular β-strand formation. This overlap is consistent with a potential role of these segments in mediating the transition between monomeric and oligomeric states. We emphasize that whereas intermolecular β-sheets are restricted to the C-terminal at 310 K, cross-β-sheet motifs in the same region are likely to emerge in longer simulations or using enhanced sampling methods.

**Figure 5:**
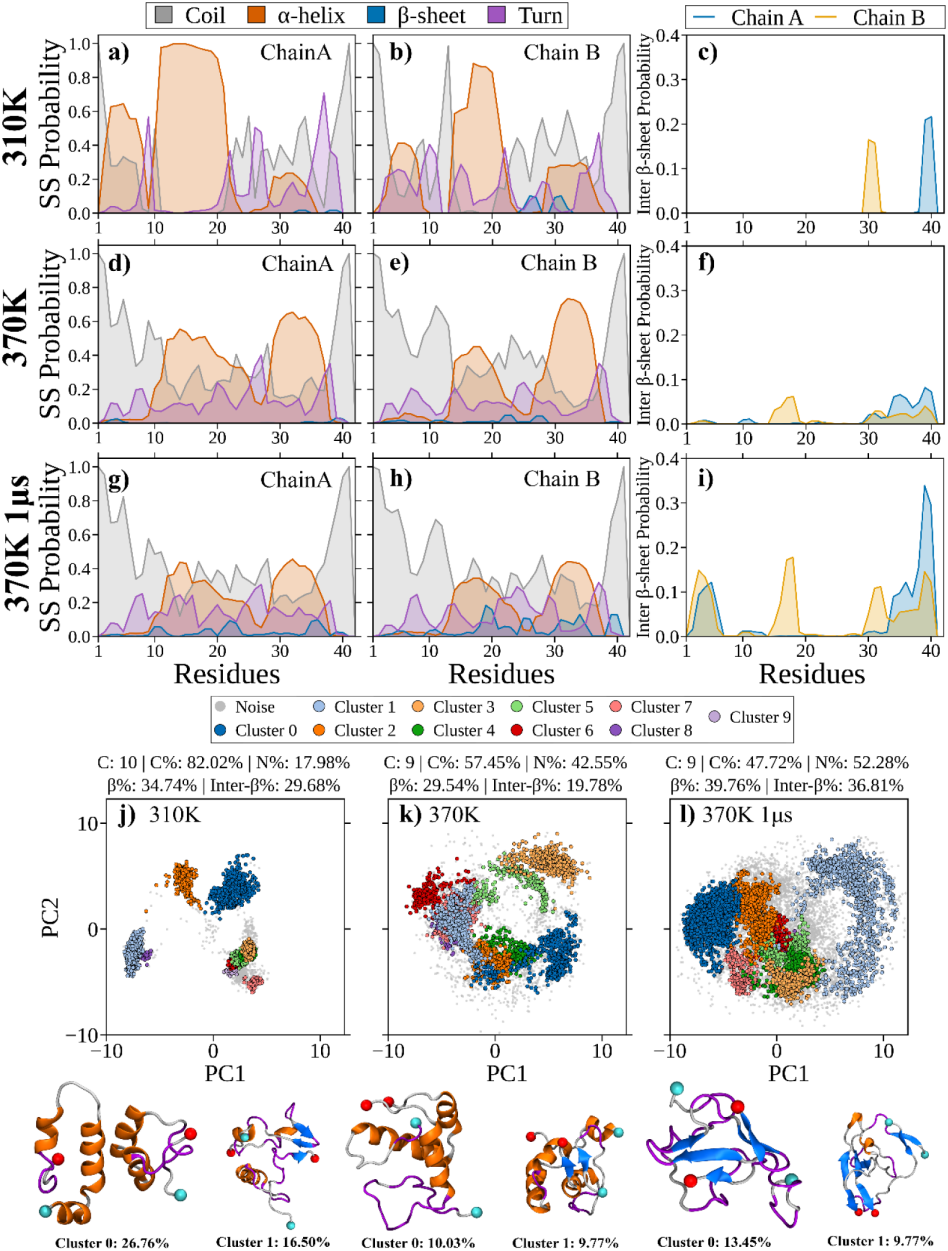
Aβ42 dimer structural properties at 310 and 370 K. (a, b) Secondary structure (intramolecular) probabilities of the monomers and (c) intermolecular (cross-) β-sheet contribution of each monomer to the dimer at 310 K; (d, e, f) similar to (a, b, c) at 370 K; (g, h, i) similar to (a, b, c) at 370 K for 1 μs trajectories; (j, k, l) PCA atomic displacement projections in the plane formed by the first two eigenvectors coupled with HDBSCAN analysis and medoid representations of the two most populated clusters (clusters 0 and 1) using 1% of the number of frames as minimal cluster size at 310, 370, and 370 K (1 μs); HDBSCAN parameters are displayed above the plots (C—number of clusters, C%—percentage of frames within clusters, N%—percentage of frames within noise, β%—percentage of frames containing β-sheet structure, inter-β%—percentage of frames containing cross-β-sheet structure.

PCA and cluster analyses reveal an enhanced dimer dynamics (i.e., a larger number of conformations) at 370 K (Figure 5j,k,l), as evidenced by the increased conformational noise. Longer trajectories show that the distribution remains broad, while its density increases, indicating persistent sampling of diverse, loosely bound intermediate states.

The medoid structures displayed in Figures 5j–l illustrate these differences: at 310 K (Figure 5j), the secondary structure of the monomers is largely preserved; at 370 K (Figure 5k), there is some α-helix loss and a progressive formation of intermolecular β-sheets and turn motifs, especially for longer simulations (Figure 5l).

Notice that although intermolecular β-sheets are more localized at 310 K the fraction at 370 K is lower for equivalent simulation times (19.8% at 370 K and 29.7% at 310 K; Figure 5k and j). This difference parallels with a substantially higher fraction of noise conformations at 370 K (42.6%) compared to 310 K (18.0%), indicating an increased conformational heterogeneity at high temperatures. Furthermore, the intermolecular β-sheets localized near the C-terminal region of the peptides at 310 K (Figure 5c) are mostly captured within cluster 1 as can be seen in the corresponding medoid structure (Figure 5j).

Longer trajectories at 370 K show an increase in the intermolecular β-sheet content and noise conformations (Figure 5l), consistent with an enhancement of the conformational space of the dimer at 370 K. This means that a less stringent clustering definition should increase the average number of β-sheet structures, particularly at 370 K, through noise reduction. This was verified by performing a similar analysis for a minimum cluster size of 0.1% of the analyzed frames (as opposed to 1%) (see Figure S3). Thus, whereas using a 1% minimum cluster size the system displays a higher β-sheet population at 310 K than at 370 K, this trend reverses (Figure S3a and b) upon reducing the noise contribution through a looser clustering definition. Furthermore, clusters 0 and 1 remain almost unchanged at 310 K (see medoid structures; see Figure S3a), as opposed to the structures at 370 K; the latter are significantly altered by the increase of the number of conformations. These results indicate that temperature accelerates the structural reorganization associated with aggregation, promoting cross-β-sheet formation and increasing the conformational freedom of the dimer. A possible increase in configurational entropy could explain why aggregation is favored over the partial collapse of the isolated monomers, where enhanced conformational freedom relative to 310 K is less marked. Figure S4 further illustrates this behavior by depicting the intramolecular and intermolecular contact maps in the dimer. A pronounced decrease of the residue–residue intermolecular distances is observed at 370 K whereas the intramolecular distances in each monomer exhibit a decrease between some residues but mostly an increase between others. Further, it can be seen that both intramolecular and intermolecular interactions involving the residues 16–22 and 29–36 play an important role in the dimer. This also reflects in the intramolecular and intermolecular HBs. Figure 6 shows the number of intramolecular HBs between the regions 16–22 and 29–36 at 310 and 370 K in the monomer and both chains of the dimer. Additionally, the intermolecular HBs between the 16–22 and 29–36 sequences (that is between 16 and 22 and 16–22 and between 16 and 22 and 29–36) in both monomers is also shown.

**Figure 6:**
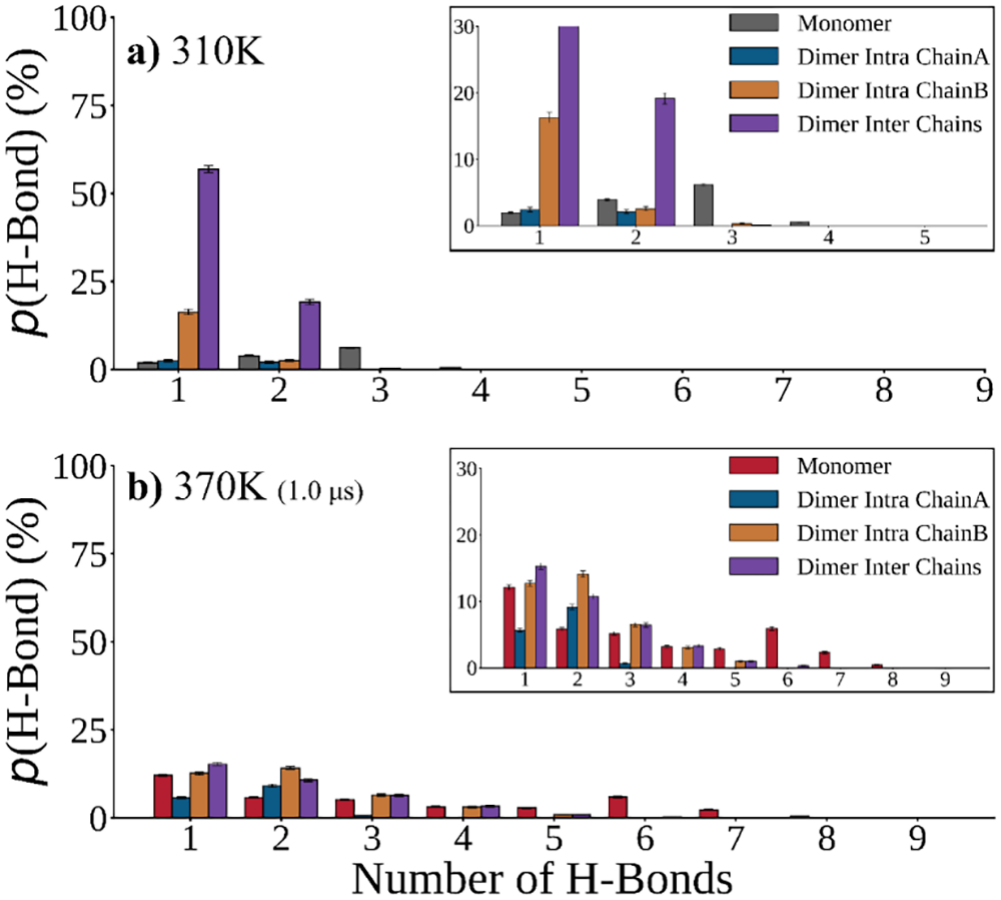
Probability of finding a conformation with *N*_HBs_ between the sequences 16–22 and 29–36 of Aβ42 at (a) 310 K and (b) 370 K. The contacts between the sequences 16–22 and 29–36 in Aβ42 were found to form a hairpin in Aβ42, but not in Aβ40.^89^ These contacts have been linked with the larger aggregation propensity of Aβ42 relative to Aβ40. Error bars are standard deviations of the probability, σ = √*p*(1 – *p*)/*N*, where *p* is the probability of observing a frame with *N*_HBs_. Results for the first 500 ns at 370 K exhibit a similar behavior; this trajectory was extended to ensure the reliability of the results.

As can be seen there is a competition between the intramolecular and intermolecular HBs involving the 16–22 and 29–36 sequences. This is especially perceptible at 370 K where the sequences 16–22 and 29–36 depict a similar number of intramolecular and intermolecular HBs.

### Aβ42 Oligomerization

To gain additional insight into the aggregation of Aβ42 we also investigated the putative aggregation of a tetramer at 310 and 370 K. Figure 7 displays the distribution of *R*_*g*_ and SASA observed for the monomer, dimer (bound state), and tetramer (bound state) at both temperatures. These results support the previous discussion in that hydrophobic interactions favor aggregation over intramolecular hydrophobic collapse, and that aggregation results in monomer elongation. However, for the tetramer, a behavior reminiscent of the quaternary structure of a globular protein can be seen, with a pronounced reduction in the SASA because of the hydrophobic collapse. Thus, although the *R*_*g*_ of the monomers increases with temperature, that of the tetramer decreases, resembling a spherical oligomer (see Figure 7j), consistent with some structures observed *in vitro*.^31,32,97^ Interestingly Khaled et al.^97^ reported that oligomers initially grow spherically but start to form extended linear aggregates at oligomeric states larger than those of the tetramer.

**Figure 7:**
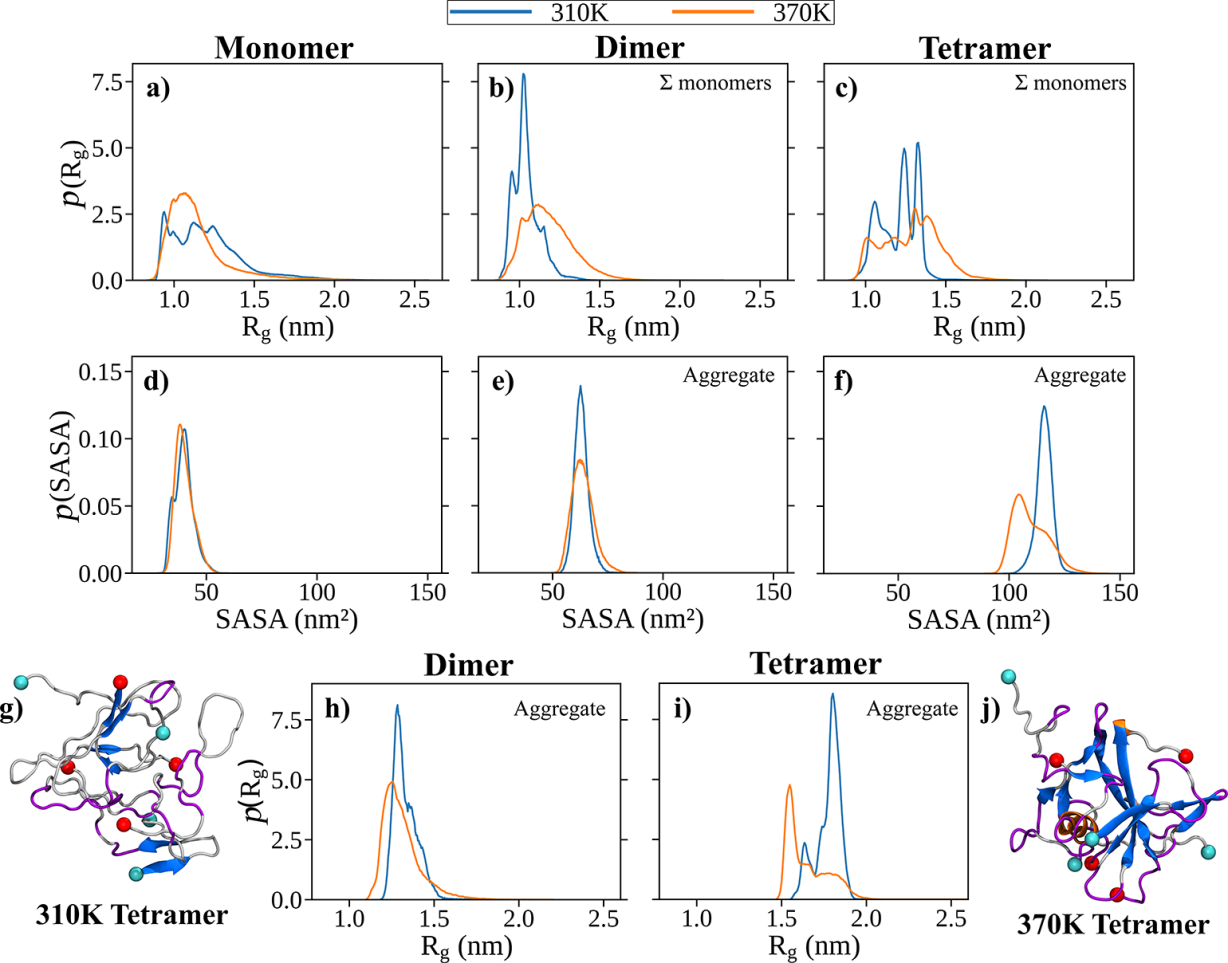
Radius of gyration (Rg) of Aβ42 at 310 and 370 K for the (a) monomer, (b) dimer, and (c) tetramer; the *R_g_* for the dimer and tetramer is the average over the different monomers and replicates. SASA of Aβ42 at 310 and 370 K for the (d) monomer, (e) dimer, and (f) tetramer; the SASA was calculated for the whole dimer and tetramer. *R_g_* for the (h) dimer and (i) tetramer; the *R_g_* was calculated for the whole dimer and tetramer. Representative snapshots of the tetramer at (g) 310 K and (j) 370 K illustrating a more spherical-like conformation at high temperatures due to increased hydrophobic interactions, despite a slight shift of the *R_g_* of the individual monomers to larger values (see (c)).

We stress, nonetheless, that although several structural features are observed both at 310 K and 370 K, aggregation pathways at high temperatures might significantly differ from those at physiological conditions.

Figure 8 shows the intermolecular (cross-) β-sheet associated with each chain in the tetramer at 310 and 370 K. As can be seen, two of the four peptides display some cross-β-sheet around the regions 16–22 and 29–36 at 370 K, that is, the same regions where the formation of intramolecular β-sheet was more evident for the monomer (see Figure 1b). At 310 K more localized regions, similar to the dimer, are observed.

**Figure 8:**
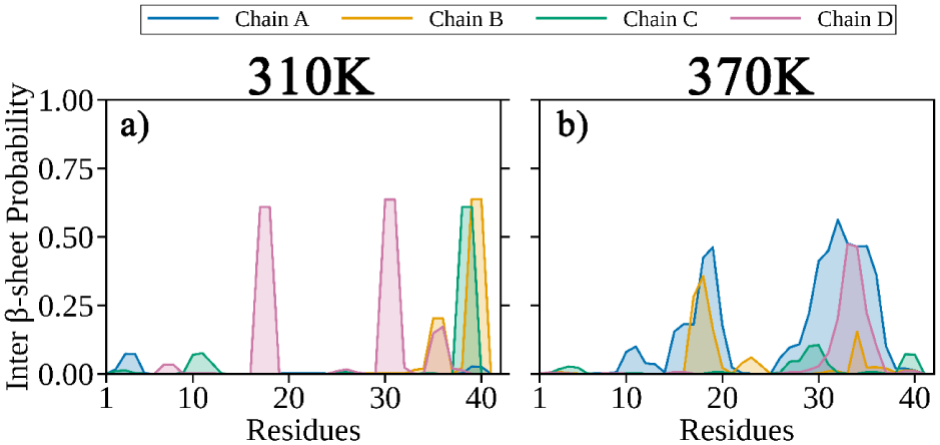
Inter (cross-) β-sheet found for the Aβ42 tetramer at 310 and 370 K. The intermolecular β-sheet was calculated for the time-windows over the different replicates where the tetramer was found in the bound state.

These results indicate that the same residues mediate intra- and intermolecular β-sheets upon temperature induced hydrophobic compaction of the monomer and aggregation of the dimer and tetramer.

## Conclusions

We studied the role of temperature on the structure of monomers of Aβ42 and α-syn and on the dimerization and oligomerization of Aβ42. Our results reproduce the experimental compaction of the Aβ42 and α-syn monomers at 370 K, while evidencing a more heterogeneous ensemble of structures and an elongation of the monomers in the dimer and tetramer. Additionally, the increase in β-sheet content observed in Aβ42 monomers at high temperatures translates into the formation of cross-β sheets between monomers in the dimer and tetramer. Furthermore, the residues that form intramolecular β-sheets in the monomer largely coincide with those involved in intermolecular HBs and cross-β motifs in the dimer and tetramer, suggesting that intramolecular β-sheet formation is intrinsically linked to aggregation propensity. Thus, upon increasing temperature, noninteracting monomers adopt more collapsed structures. However, below some intermolecular distance (or above some concentration), β-sheets arising from intramolecular HBs transform into intermolecular cross-β sheets, promoting both a reduction in SASA and *R*_*g*_, reflecting the formation of spherical-like shape (reminiscent of the folding of globular protein) whereas the monomers undergo elongation in average. Whether the formation of more compact monomers with increased β-sheet content is a necessary condition for aggregation remains unclear. Although this possibility cannot be excluded, our results provide no evidence that such a structural transformation of the monomer is either necessary or sufficient for aggregation to occur. This study demonstrates that several key experimental features of the monomer and protofibrils are well reproduced at high temperatures, where the enhanced hydrophobic effect facilitates protein collapse and aggregation on accelerated time scales.

## Supplementary Material



## Notes

Number of intermolecular contacts between the two Aβ42 monomers and between the 16–22 region at 310 and 370 K; number of intermolecular contacts between the two Aβ42 monomers for different contact definitions; PCA atomic displacement projections of the Aβ42 dimer in the plane formed by the first two eigenvectors combined with HDBSCAN analysis; average intramolecular and intermolecular distances between all C_α_ pairs in the Aβ42 dimer at 310 and 370 K (PDF)

## References

[1] Lambert M. P., Barlow A. K., Chromy B. A., Edwards C., Freed R., Liosatos M., Morgan T. E., Rozovsky I., Trommer B., Viola K. L., Wals P., Zhang C., Finch C. E., Krafft G. A., Klein W. L. D. (1998). Nonfibrillar Ligands Derived from
Aβ_1–42_ Are Potent Central Nervous System Neurotoxins. Proc. Natl. Acad. Sci. U. S. A..

[2] McLean C. A., Cherny R. A., Fraser F. W., Fuller S. J., Smith M. J., Vbeyreuther K., Bush A. I., Masters C. L. (1999). Soluble pool of
A? amyloid as a determinant of severity of neurodegeneration in Alzheimer’s
disease. Ann. Neurol..

[3] Bucciantini M., Giannoni E., Chiti F., Baroni F., Formigli L., Zurdo J., Taddei N., Ramponi G., Dobson C. M., Stefani M. (2002). Inherent Toxicity of
Aggregates Implies a Common Mechanism
for Protein Misfolding Diseases. Nature.

[4] Walsh D. M., Klyubin I., Fadeeva J. V., Cullen W. K., Anwyl R., Wolfe M. S., Rowan M. J., Selkoe D. J. (2002). Naturally Secreted
Oligomers of Amyloid β Protein Potently Inhibit Hippocampal
Long-Term Potentiation in Vivo. Nature.

[5] Tang H., Andrikopoulos N., Li Y., Ke S., Sun Y., Ding F., Ke P. C. (2025). Emerging
Biophysical Origins and
Pathogenic Implications of Amyloid Oligomers. Nat. Commun..

[6] Walsh D. M., Selkoe D. J. (2007). Aβ Oligomers – a Decade of Discovery. J. Neurochem..

[7] Cline E. N., Bicca M. A., Viola K. L., Klein W. L. (2018). The Amyloid-β
Oligomer Hypothesis: Beginning of the Third Decade. JAD.

[8] Winner B., Jappelli R., Maji S. K., Desplats P. A., Boyer L., Aigner S., Hetzer C., Loher T., Vilar M., Campioni S., Tzitzilonis C., Soragni A., Jessberger S., Mira H., Consiglio A., Pham E., Masliah E., Gage F. H., Riek R. (2011). In Vivo Demonstration That α-Synuclein
Oligomers Are Toxic. Proc. Natl. Acad. Sci.
U. S. A..

[9] Ono K. (2017). The Oligomer
Hypothesis in α-Synucleinopathy. Neurochem.
Res..

[10] Gong C.-X., Liu F., Iqbal K. (2018). Multifactorial Hypothesis
and Multi-Targets for Alzheimer’s
Disease. JAD.

[11] Duchesne S., Rousseau L.-S., Belzile-Marsolais F., Welch L.-A., Cournoyer B., Arseneau M., Lapierre V., Poulin S.-M., Potvin O., Hudon C. (2024). A Scoping Review of
Alzheimers Disease Hypotheses: An Array of Uni-
and Multi-Factorial Theories. J. Alzheimer’s
Dis..

[12] Parker J., Moris J. M., Goodman L. C., Paidisetty V. K., Vanegas V., Turner H. A., Melgar D., Koh Y. (2025). A Multifactorial
Lens on Risk Factors Promoting the Progression of Alzheimer’s
Disease. Brain Res..

[13] Selkoe D. J. (2002). Alzheimer’s
Disease Is a Synaptic Failure. Science.

[14] Kauzmann, W. Some Factors in the Interpretation of Protein Denaturation. In Advances in Protein Chemistry; Elsevier, 1959; Vol. 14, pp. 1–63. DOI: 10.1016/S0065-3233(08)60608-7. 14404936

[15] Tanford C. (1978). The Hydrophobic
Effect and the Organization of Living Matter. Science.

[16] Dill K. A. (1990). Dominant
Forces in Protein Folding. Biochemistry.

[17] Dill K. A., MacCallum J. L. (2012). The Protein-Folding Problem, 50 Years On. Science.

[18] Chandler D. (2005). Interfaces
and the Driving Force of Hydrophobic Assembly. Nature.

[19] Chiti F., Dobson C. M. (2017). Protein Misfolding, Amyloid Formation, and Human Disease:
A Summary of Progress Over the Last Decade. Annu. Rev. Biochem..

[20] Sawaya M. R., Hughes M. P., Rodriguez J. A., Riek R., Eisenberg D. S. (2021). The Expanding
Amyloid Family: Structure, Stability, Function, and Pathogenesis. Cell.

[21] Stefani M. (2010). Structural
Polymorphism of Amyloid Oligomers and Fibrils Underlies Different
Fibrillization Pathways: Immunogenicity and Cytotoxicity. CPPS.

[22] Stefani M. (2012). Structural
Features and Cytotoxicity of Amyloid Oligomers: Implications in Alzheimer’s
Disease and Other Diseases with Amyloid Deposits. Prog. Neurobiol..

[23] Paravastu A. K., Leapman R. D., Yau W.-M., Tycko R. (2008). Molecular Structural
Basis for Polymorphism in Alzheimer’s β-Amyloid Fibrils. Proc. Natl. Acad. Sci. U. S. A..

[24] Lu J.-X., Qiang W., Yau W.-M., Schwieters C. D., Meredith S. C., Tycko R. (2013). Molecular Structure
of β-Amyloid
Fibrils in Alzheimer’s Disease Brain Tissue. Cell.

[25] Colvin M. T., Silvers R., Ni Q. Z., Can T. V., Sergeyev I., Rosay M., Donovan K. J., Michael B., Wall J., Linse S., Griffin R. G. (2016). Atomic Resolution
Structure of Monomorphic
Aβ _42_ Amyloid Fibrils. J. Am.
Chem. Soc..

[26] Gremer L., Schölzel D., Schenk C., Reinartz E., Labahn J., Ravelli R. B. G., Tusche M., Lopez-Iglesias C., Hoyer W., Heise H., Willbold D., Schröder G. F. (2017). Fibril
Structure of Amyloid-β(1–42) by Cryo–Electron
Microscopy. Science.

[27] Yang Y., Arseni D., Zhang W., Huang M., Lövestam S., Schweighauser M., Kotecha A., Murzin A. G., Peak-Chew S. Y., Macdonald J., Lavenir I., Garringer H. J., Gelpi E., Newell K. L., Kovacs G. G., Vidal R., Ghetti B., Ryskeldi-Falcon B., Scheres S. H. W., Goedert M. (2022). Cryo-EM Structures
of Amyloid-β 42 Filaments from Human Brains. Science.

[28] Lee M., Yau W.-M., Louis J. M., Tycko R. (2023). Structures of Brain-Derived
42-Residue Amyloid-β Fibril Polymorphs with Unusual Molecular
Conformations and Intermolecular Interactions. Proc. Natl. Acad. Sci. U. S. A..

[29] Miller Y., Ma B., Nussinov R. (2010). Polymorphism
in Alzheimer Aβ Amyloid Organization
Reflects Conformational Selection in a Rugged Energy Landscape. Chem. Rev..

[30] Barghorn S., Nimmrich V., Striebinger A., Krantz C., Keller P., Janson B., Bahr M., Schmidt M., Bitner R. S., Harlan J., Barlow E., Ebert U., Hillen H. (2005). Globular Amyloid
Β-peptide_1–42_ Oligomer – a Homogenous
and Stable Neuropathological Protein in Alzheimer’s Disease. J. Neurochem..

[31] Hoshi M., Sato M., Matsumoto S., Noguchi A., Yasutake K., Yoshida N., Sato K. (2003). Spherical
Aggregates of β-Amyloid
(Amylospheroid) Show High Neurotoxicity and Activate Tau Protein Kinase
I/Glycogen Synthase Kinase-3β. Proc. Natl.
Acad. Sci. U. S. A..

[32] Noguchi A., Matsumura S., Dezawa M., Tada M., Yanazawa M., Ito A., Akioka M., Kikuchi S., Sato M., Ideno S., Noda M., Fukunari A., Muramatsu S., Itokazu Y., Sato K., Takahashi H., Teplow D. B., Nabeshima Y., Kakita A., Imahori K., Hoshi M. (2009). Isolation and Characterization of Patient-Derived, Toxic, High Mass
Amyloid β-Protein (Aβ) Assembly from Alzheimer Disease
Brains. J. Biol. Chem..

[33] Stroud J. C., Liu C., Teng P. K., Eisenberg D. (2012). Toxic Fibrillar Oligomers of Amyloid-β
Have Cross-β Structure. Proc. Natl. Acad.
Sci. U. S. A..

[34] Wu J. W., Breydo L., Isas J. M., Lee J., Kuznetsov Y. G., Langen R., Glabe C. (2010). Fibrillar Oligomers
Nucleate the
Oligomerization of Monomeric Amyloid β but Do Not Seed Fibril
Formation. J. Biol. Chem..

[35] Österlund N., Moons R., Ilag L. L., Sobott F., Gräslund A. (2019). Native Ion
Mobility-Mass Spectrometry Reveals the Formation of β-Barrel
Shaped Amyloid-β Hexamers in a Membrane-Mimicking Environment. J. Am. Chem. Soc..

[36] Ciudad S., Puig E., Botzanowski T., Meigooni M., Arango A. S., Do J., Mayzel M., Bayoumi M., Chaignepain S., Maglia G., Cianferani S., Orekhov V., Tajkhorshid E., Bardiaux B., Carulla N. (2020). Aβ­(1–42)
Tetramer and
Octamer Structures Reveal Edge Conductivity Pores as a Mechanism for
Membrane Damage. Nat. Commun..

[37] Ahmed M., Davis J., Aucoin D., Sato T., Ahuja S., Aimoto S., Elliott J. I., Van Nostrand W. E., Smith S. O. (2010). Structural Conversion of Neurotoxic
Amyloid-Β1–42
Oligomers to Fibrils. Nat. Struct. Mol. Biol..

[38] Yu L., Edalji R., Harlan J. E., Holzman T. F., Lopez A. P., Labkovsky B., Hillen H., Barghorn S., Ebert U., Richardson P. L., Miesbauer L., Solomon L., Bartley D., Walter K., Johnson R. W., Hajduk P. J., Olejniczak E. T. (2009). Structural
Characterization of a Soluble Amyloid β-Peptide Oligomer. Biochemistry.

[39] Kayed R., Pensalfini A., Margol L., Sokolov Y., Sarsoza F., Head E., Hall J., Glabe C. (2009). Annular Protofibrils
Are a Structurally and Functionally Distinct Type of Amyloid Oligomer. J. Biol. Chem..

[40] Ono K., Condron M. M., Teplow D. B. (2009). Structure–Neurotoxicity
Relationships
of Amyloid β-Protein Oligomers. Proc.
Natl. Acad. Sci. U. S. A..

[41] Volles M. J., Lansbury P. T. (2002). Vesicle Permeabilization
by Protofibrillar α-Synuclein
Is Sensitive to Parkinson’s Disease-Linked Mutations and Occurs
by a Pore-like Mechanism. Biochemistry.

[42] Cremades N., Cohen S. I. A., Deas E., Abramov A. Y., Chen A. Y., Orte A., Sandal M., Clarke R. W., Dunne P., Aprile F. A., Bertoncini C. W., Wood N. W., Knowles T. P. J., Dobson C. M., Klenerman D. (2012). Direct Observation
of the Interconversion
of Normal and Toxic Forms of α-Synuclein. Cell.

[43] Danzer K. M., Haasen D., Karow A. R., Moussaud S., Habeck M., Giese A., Kretzschmar H., Hengerer B., Kostka M. (2007). Different
Species of α-Synuclein Oligomers Induce Calcium Influx and Seeding. J. Neurosci..

[44] Curtain C. C., Kirby N. M., Mertens H. D. T., Barnham K. J., Knott R. B., Masters C. L., Cappai R., Rekas A., Kenche V. B., Ryan T. (2014). Alpha-Synuclein Oligomers
and Fibrils Originate in Two Distinct Conformer
Pools: A Small Angle X-Ray Scattering and Ensemble Optimisation Modelling
Study. Mol. BioSyst..

[45] Liu P., Reed M. N., Kotilinek L. A., Grant M. K. O., Forster C. L., Qiang W., Shapiro S. L., Reichl J. H., Chiang A. C. A., Jankowsky J. L., Wilmot C. M., Cleary J. P., Zahs K. R., Ashe K. H. (2015). Quaternary
Structure Defines a Large Class of Amyloid-β
Oligomers Neutralized by Sequestration. Cell
Rep..

[46] Arispe N., Rojas E., Pollard H. B. (1993). Alzheimer Disease
Amyloid Beta Protein
Forms Calcium Channels in Bilayer Membranes: Blockade by Tromethamine
and Aluminum. Proc. Natl. Acad. Sci. U. S. A..

[47] Glabe C. G., Kayed R. (2006). Common Structure and Toxic Function of Amyloid Oligomers Implies
a Common Mechanism of Pathogenesis. Neurology.

[48] Wuttke R., Hofmann H., Nettels D., Borgia M. B., Mittal J., Best R. B., Schuler B. (2014). Temperature-Dependent
Solvation Modulates
the Dimensions of Disordered Proteins. Proc.
Natl. Acad. Sci. U. S. A..

[49] Uversky V. N. (2009). Intrinsically
Disordered Proteins and Their Environment: Effects of Strong Denaturants,
Temperature, pH, Counter Ions, Membranes, Binding Partners, Osmolytes,
and Macromolecular Crowding. Protein J..

[50] Nettels D., Müller-Späth S., Küster F., Hofmann H., Haenni D., Rüegger S., Reymond L., Hoffmann A., Kubelka J., Heinz B., Gast K., Best R. B., Schuler B. (2009). Single-Molecule Spectroscopy
of the Temperature-Induced Collapse of Unfolded Proteins. Proc. Natl. Acad. Sci. U. S. A..

[51] Uversky V. N., Li J., Fink A. L. (2001). Evidence
for a Partially Folded Intermediate in α-Synuclein
Fibril Formation. J. Biol. Chem..

[52] Ferrone F. A. (2015). Assembly
of Aβ Proceeds via Monomeric Nuclei. J.
Mol. Biol..

[53] Sadqi M., Lapidus L. J., Muñoz V. (2003). How Fast Is
Protein Hydrophobic Collapse?. Proc. Natl. Acad.
Sci. U. S. A..

[54] Tamoliu̅nas K., Galamba N. P. D. (2020). Protein Denaturation,
Zero Entropy Temperature, and
the Structure of Water around Hydrophobic and Amphiphilic Solutes. J. Phys. Chem. B.

[55] Lum K., Chandler D., Weeks J. D. (1999). Hydrophobicity at Small and Large
Length Scales. J. Phys. Chem. B.

[56] Berne B. J., Weeks J. D., Zhou R. (2009). Dewetting
and Hydrophobic Interaction
in Physical and Biological Systems. Annu. Rev.
Phys. Chem..

[57] Huang D. M., Chandler D. (2000). Temperature and Length
Scale Dependence of Hydrophobic
Effects and Their Possible Implications for Protein Folding. Proc. Natl. Acad. Sci. U. S. A..

[58] Southall N. T., Dill K. A., Haymet A. D. J. (2002). A View
of the Hydrophobic Effect. J. Phys. Chem. B.

[59] Galamba N. (2021). Free Energy
Convergence in Short- and Long-Length Hydrophobic Hydration. J. Mol. Liq..

[60] Muñoz V., Thompson P. A., Hofrichter J., Eaton W. A. (1997). Folding Dynamics
and Mechanism of β-Hairpin Formation. Nature.

[61] Kjaergaard M., Nørholm A., Hendus–Altenburger R., Pedersen S. F., Poulsen F. M., Kragelund B. B. (2010). Temperature-dependent structural
changes in intrinsically disordered proteins: Formation of α–helices
or loss of polyproline II?. Protein Sci..

[62] Battisti A., Ciasca G., Grottesi A., Tenenbaum A. (2017). Thermal Compaction
of the Intrinsically Disordered Protein Tau: Entropic, Structural,
and Hydrophobic Factors. Phys. Chem. Chem. Phys..

[63] Tomaselli S., Esposito V., Vangone P., Van Nuland N. A. J., Bonvin A. M. J. J., Guerrini R., Tancredi T., Temussi P. A., Picone D. (2006). The Α-to-β
Conformational
Transition of Alzheimer’s Aβ-(1–42) Peptide in
Aqueous Media Is Reversible: A Step by Step Conformational Analysis
Suggests the Location of β Conformation Seeding. ChemBioChem.

[64] Martins G. F., Rocha C., Galamba N. (2025). Protein Recognition of Linear and
Cyclic Peptides of Homologous Sequences Implicated in the Aggregation
of α-Synuclein. J. Phys. Chem. B.

[65] Jephthah S., Staby L., Kragelund B. B., Skepö M. (2019). Temperature
Dependence of Intrinsically Disordered Proteins in Simulations: What
Are We Missing?. J. Chem. Theory Comput..

[66] Hicks A., Zhou H.-X. (2018). Temperature-Induced Collapse of a Disordered Peptide
Observed by Three Sampling Methods in Molecular Dynamics Simulations. J. Chem. Phys..

[67] Huang J., Rauscher S., Nawrocki G., Ran T., Feig M., De Groot B. L., Grubmüller H., MacKerell A. D. (2017). CHARMM36m:
An Improved Force Field for Folded and Intrinsically Disordered Proteins. Nat. Methods.

[68] Xiao Y., Ma B., McElheny D., Parthasarathy S., Long F., Hoshi M., Nussinov R., Ishii Y. (2015). Aβ­(1–42) Fibril Structure
Illuminates Self-Recognition and Replication of Amyloid in Alzheimer’s
Disease. Nat. Struct. Mol. Biol..

[69] Tuttle M. D., Comellas G., Nieuwkoop A. J., Covell D. J., Berthold D. A., Kloepper K. D., Courtney J. M., Kim J. K., Barclay A. M., Kendall A., Wan W., Stubbs G., Schwieters C. D., Lee V. M. Y., George J. M., Rienstra C. M. (2016). Solid-State NMR
Structure of a Pathogenic Fibril of Full-Length Human α-Synuclein. Nat. Struct. Mol. Biol..

[70] Kabsch W., Sander C. (1983). Dictionary of Protein
Secondary Structure: Pattern
Recognition of Hydrogen-bonded and Geometrical Features. Biopolymers.

[71] Van D. S., Lindahl E., Hess B., Groenhof G., Mark G. A. E., Berendsen H. J. C. (2005). GROMACS:
Fast, Flexible, and Free. J. Comput. Chem..

[72] Evans D. J., Holian B. L. (1985). The Nose–Hoover
Thermostat. J. Chem. Phys..

[73] Hoover W. G. (1985). Canonical
Dynamics: Equilibrium Phase-Space Distributions. Phys. Rev. A.

[74] Parrinello M., Rahman A. (1981). Polymorphic Transitions in Single
Crystals: A New Molecular
Dynamics Method. J. Appl. Phys..

[75] Essmann U., Perera L., Berkowitz M. L., Darden T., Lee H., Pedersen L. G. (1995). A Smooth Particle
Mesh Ewald Method. J. Chem. Phys..

[76] Hess B., Bekker H., Berendsen H. J. C., Fraaije J. G. E. M. (1997). LINCS: A Linear
Constraint Solver for Molecular Simulations. J. Comput. Chem..

[77] Amadei A., Linssen A. B., Berendsen H. J. (1993). Essential
Dynamics of Proteins. Proteins.

[78] Campello, R. J. G. B. ; Moulavi, D. ; Sander, J. Density-Based Clustering Based on Hierarchical Density Estimates. In Advances in Knowledge Discovery and Data Mining; Pei, J. ; Tseng, V. S. ; Cao, L. ; Motoda, H. ; Xu, G. , Eds.; Density-Based Clustering Based on Hierarchical Density Estimates, In Advances in Knowledge Discovery and Data Mining; Springer: Berlin Heidelberg, 2013, Vol. 7819, pp. 160–172. DOI: 10.1007/978-3-642-37456-2_14.

[79] Balsera M. A., Wriggers W., Oono Y., Schulten K. (1996). Principal Component
Analysis and Long Time Protein Dynamics. J.
Phys. Chem..

[80] Gomes, I. ; Martins, G. ; Galamba, N. Essential Dynamics of Ubiquitin in Water and in a Natural Deep Eutectic Solvent,ChemRxiv, 2024, 10.26434/chemrxiv-2024-7l988. 38904333

[81] Berendsen H. (2000). Collective
Protein Dynamics in Relation to Function. Curr.
Opin. Struct. Biol..

[82] Martins G. F., Galamba N. (2024). Wild-Type α-Synuclein Structure
and Aggregation:
A Comprehensive Coarse-Grained and All-Atom Molecular Dynamics Study. J. Chem. Inf. Model..

[83] Melvin R. L., Godwin R. C., Xiao J., Thompson W. G., Berenhaut K. S., Salsbury F. R. (2016). Uncovering Large-Scale Conformational Change in Molecular
Dynamics without Prior Knowledge. J. Chem. Theory
Comput..

[84] Bhattacharya S., Chakrabarty S. (2025). Mapping Conformational Landscape
in Protein Folding:
Benchmarking Dimensionality Reduction and Clustering Techniques on
the Trp-Cage Mini-Protein. Biophys. Chem..

[85] Prim R. C. (1957). Shortest
Connection Networks And Some Generalizations. Bell Syst. Tech. J..

[86] Baldwin R. L. (1986). Temperature
Dependence of the Hydrophobic Interaction in Protein Folding. Proc. Natl. Acad. Sci. U. S. A..

[87] Balbach J. J., Ishii Y., Antzutkin O. N., Leapman R. D., Rizzo N. W., Dyda F., Reed J., Tycko R. (2000). Amyloid Fibril Formation
by Aβ_16–22_, a Seven-Residue Fragment of the
Alzheimer’s β-Amyloid Peptide, and Structural Characterization
by Solid State NMR. Biochemistry.

[88] Favrin G., Irbäck A., Mohanty S. (2004). Oligomerization of Amyloid Aβ16–22
Peptides Using Hydrogen Bonds and Hydrophobicity Forces. Biophys. J..

[89] Ball K. A., Phillips A. H., Wemmer D. E., Head-Gordon T. (2013). Differences
in β-Strand Populations of Monomeric Aβ40 and Aβ42. Biophys. J..

[90] Lührs T., Ritter C., Adrian M., Riek-Loher D., Bohrmann B., Döbeli H., Schubert D., Riek R. (2005). 3D Structure
of Alzheimer’s Amyloid-β(1–42) Fibrils. Proc. Natl. Acad. Sci. U. S. A..

[91] Thole J. F., Waudby C. A., Pielak G. J. (2023). Disordered
Proteins Mitigate the
Temperature Dependence of Site-Specific Binding Free Energies. J. Biol. Chem..

[92] Zerze G. H., Best R. B., Mittal J. (2015). Sequence- and Temperature-Dependent
Properties of Unfolded and Disordered Proteins from Atomistic Simulations. J. Phys. Chem. B.

[93] Chen C., Yan Z.-S., Ma Y.-Q., Ding H.-M. (2023). Effect of Terahertz
Waves on the Structure of the Aβ42 Monomer, Dimer, and Protofibril:
Insights from Molecular Dynamics Simulations. ACS Chem. Neurosci..

[94] Bertoncini C. W., Jung Y.-S., Fernandez C. O., Hoyer W., Griesinger C., Jovin T. M., Zweckstetter M. (2005). Release of
Long-Range Tertiary Interactions
Potentiates Aggregation of Natively Unstructured α-Synuclein. Proc. Natl. Acad. Sci. U. S. A..

[95] Doherty C. P. A., Ulamec S. M., Maya-Martinez R., Good S. C., Makepeace J., Khan G. N., Van Oosten-Hawle P., Radford S. E., Brockwell D. J. (2020). A Short
Motif in the N-Terminal Region of α-Synuclein Is Critical for
Both Aggregation and Function. Nat. Struct.
Mol. Biol..

[96] Krone M. G., Hua L., Soto P., Zhou R., Berne B. J., Shea J.-E. (2008). Role of
Water in Mediating the Assembly of Alzheimer Amyloid-β Aβ16–22
Protofilaments. J. Am. Chem. Soc..

[97] Khaled M., Rönnbäck I., Ilag L. L., Gräslund A., Strodel B., Österlund N. (2023). A Hairpin
Motif in the Amyloid-β
Peptide Is Important for Formation of Disease-Related Oligomers. J. Am. Chem. Soc..

