# Supplementary material for: Temperature-Dependent Dynamics of Aβ42 and α‑Synuclein Monomers and Early Oligomerization of Aβ42: Shared Residues Mediate Intra- and Intermolecular β‑Sheets
Source: ACS Chem Neurosci. 2026 Jun 23;17(13):2504–16. doi: 10.1021/acschemneuro.6c00242 (PMC13329896; doi:10.1021/acschemneuro.6c00242)
Supplement: Supplementary file 1 [file cn6c00242_si_001.pdf]

Temperature-Dependent Dynamics of Aβ42 and α-Synuclein Monomers and Early Oligomerization of Aβ42: Shared Residues Mediate Intra- and Intermolecular β-Sheets

Supporting Information

Gabriel F. Martins[a], Cristiano Rocha[a], Nuno Galamba[a*]

[a] BioISI - Biosystems and Integrative Sciences Institute, Faculty of Sciences of the University of Lisbon, C8, Campo Grande, 1749-016 Lisbon, Portugal.

[*]Corresponding author: njgalamba@fc.ul.pt

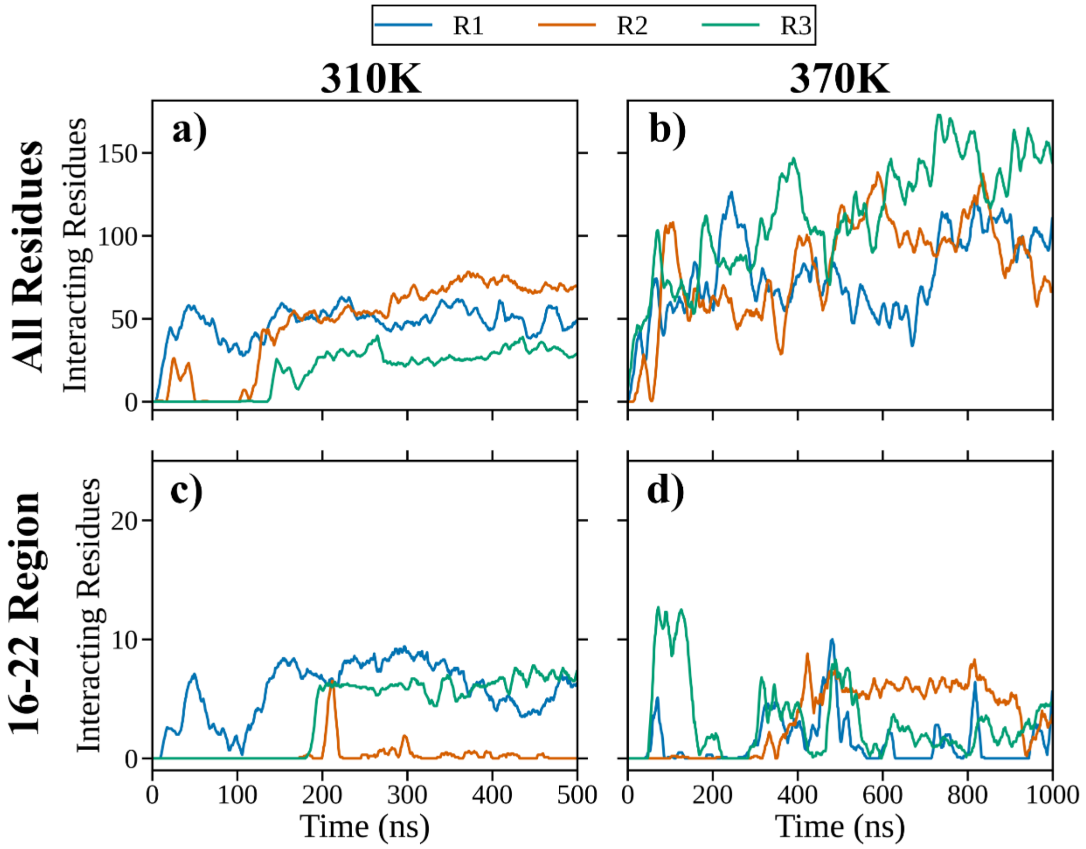

e)

System Temperature	Replica	Starting Time of Dimer Formation
	1	20ns
310K	2	140ns
	3	180ns
	1	60ns
370K	2	80ns
	3	50ns

Figure S1: Absolute number of contacts between the two Aβ42 monomers at **(a)** 310 K and **(b)** 370 K and between the 16-22 region at **(c)** 310 K and **(d)** 370 K. A contact is defined when the distance between any two heavy atoms belonging to two amino acids is less than 0.5 nm. Faster dimerization and a greater number of contacts are observed at 370 K, associated with an increased diffusion coefficient of the peptides and the hydrophobic effect. The starting time of dimer formation **(e)** marks, respectively, the end and start of the *unbound* and *bound* time windows used to analyze the peptides' structure.

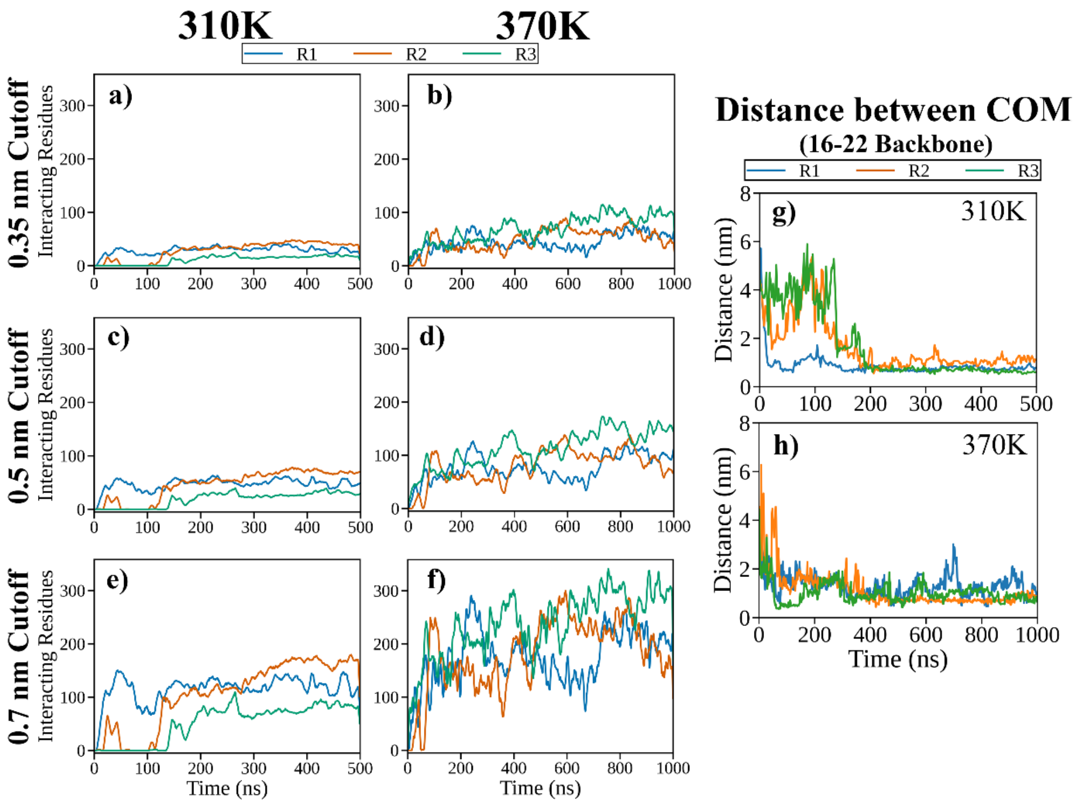

Figure S2: Absolute number of contacts between the two Aβ42 monomers for different contact definitions at **(a, c, e)** 310 K and **(b, d, f)** 370 K. A contact is defined when the distance between any two heavy atoms belonging to two amino acids is less than r_c (r_c = 0.35, 0.5, and 0.7 nm). The distance between the center-of-mass (COM) of the the backbone atoms of residues 16-22 at (**g**) 310 K and (**h**) 370 K.

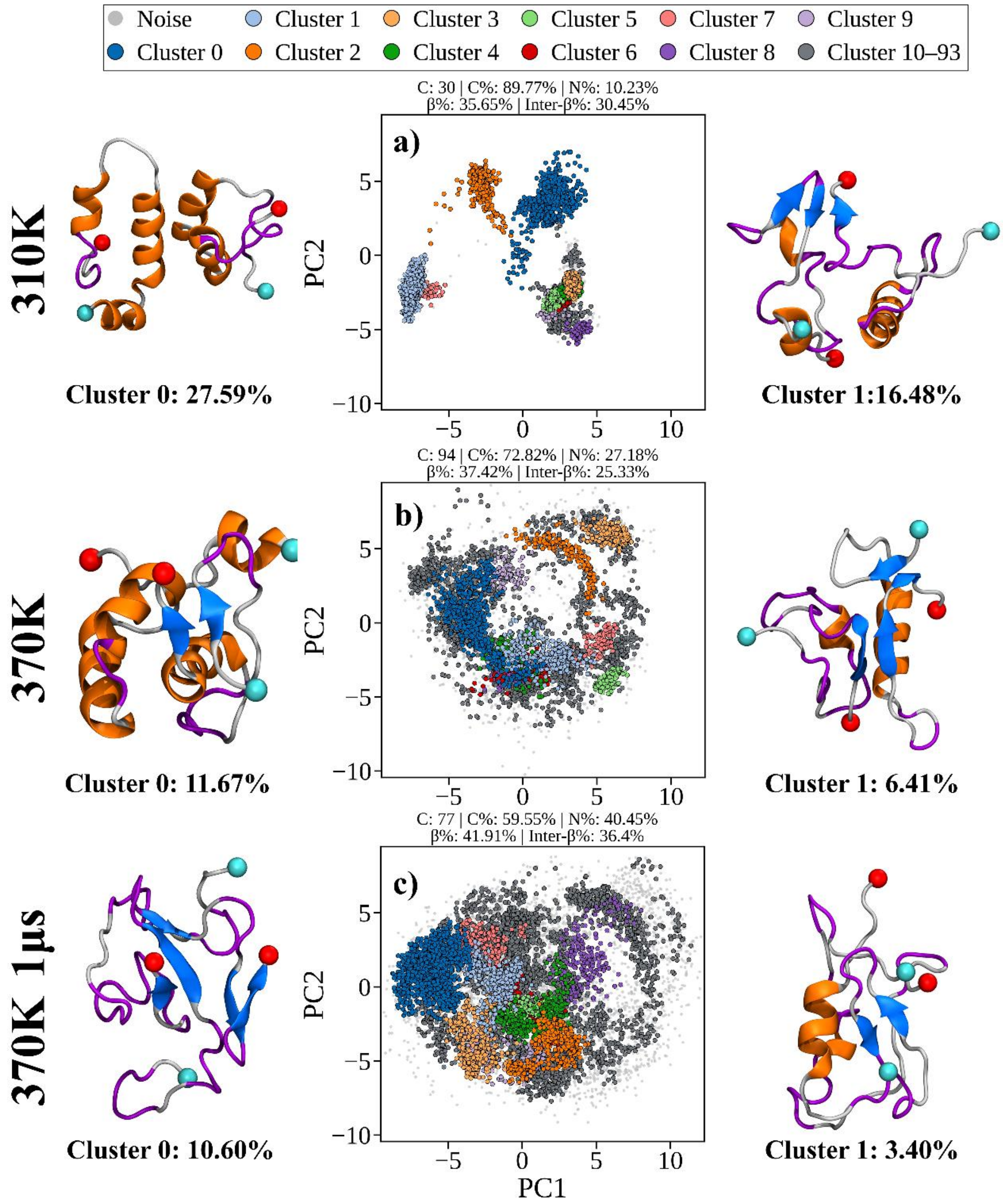

Figure S3: PCA atomic displacement projections of the Aβ42 dimer in the plane formed by the first two eigenvectors combined with HDSCAN analysis and medoid representations of the two most populated clusters for the system at **(a)** 310K, **(b)** 370K, and **(c)** 370K 1μs using 0.1% of the number of frames as minimal cluster size.

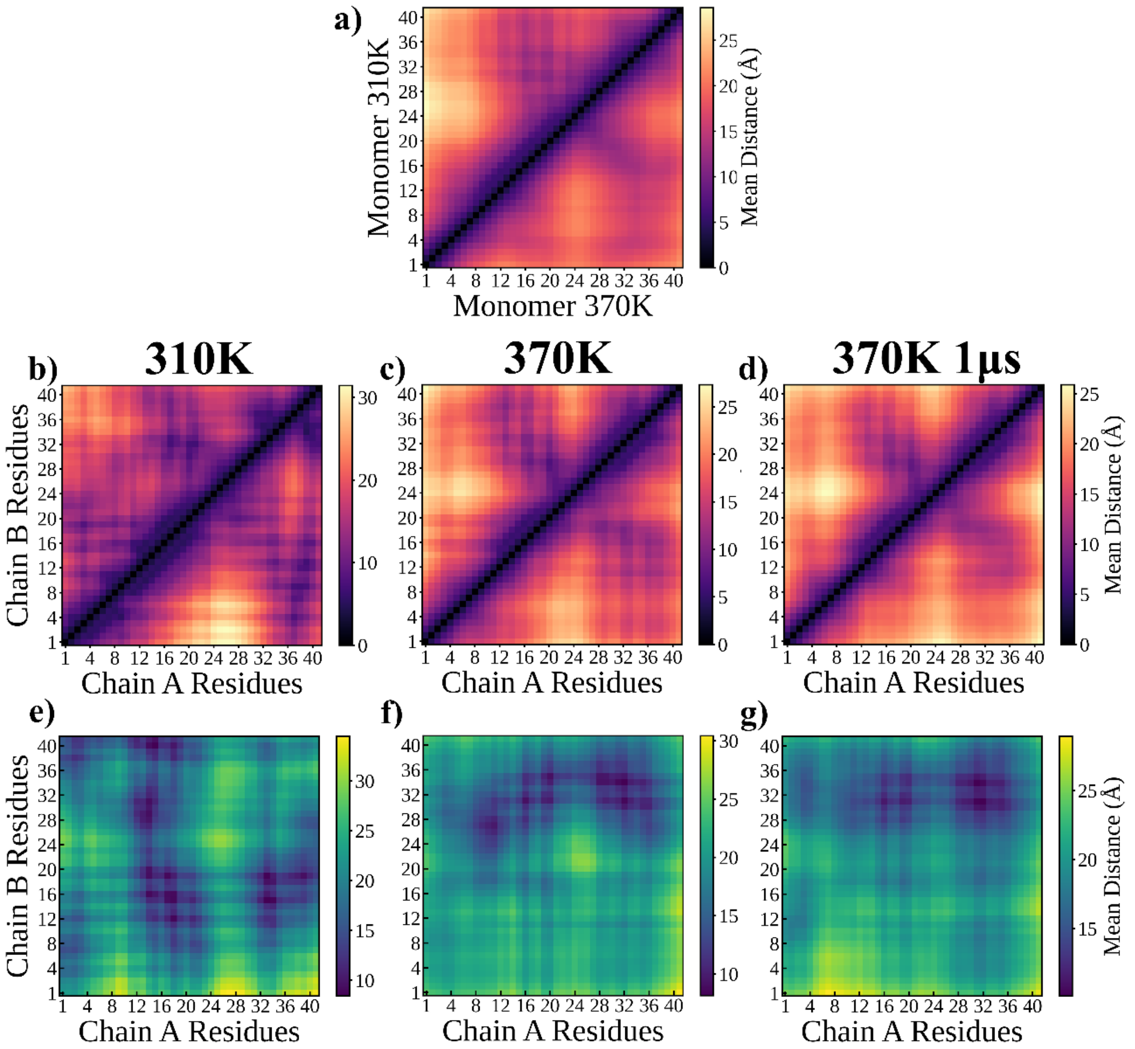

Figure S4: Average intramolecular distances between all C_α pairs in the Aβ42 monomer at (a) 310 K and (b) 370 K. Average intramolecular distances between all C_α pairs of each monomer (chain A and chain B) in the Aβ42 dimer at **(a)** 310K, **(b)** 370K, and **(c)** 370K 1μs. Average intermolecular distances between all C_α residues of both monomers in the Aβ42 dimer at **(d)** 310K **(e)** 370K, and **(f)** 370K 1μs. Pease notice the different distance scales (in units of Å) at 310 K and 370 K.
